# The role of skin mechanics in contact force variation under different friction conditions

**DOI:** 10.1038/s41598-026-41781-z

**Published:** 2026-03-01

**Authors:** İsmail Devecioğlu, Raiyaan Ruhi, Naqash Afzal, Alastair J. Loutit, Alwin So, Michaël Wiertlewski, Richard M. Vickery, Ingvars Birznieks

**Affiliations:** 1 School of Biomedical Sciences, UNSW Sydney, Randwick, NSW 2052 Australia; 2 Neuroscience Research Australia, Sydney, NSW Australia; 3 School of Engineering Mathematics and Technology, University of Bristol, Bristol, UK; 4 Department of Basic Neuroscience, Université de Genève, Geneva, Switzerland; 5 Department of Cognitive Robotics, Delft University of Technology, Delft, The Netherlands; 6 Bionics and Biorobotics, Tyree Foundation Institute of Health Engineering, UNSW Sydney, Sydney, NSW Australia

**Keywords:** Friction, Grip force control, Contact kinematics, Skin mechanics, Touch, Slipperiness, Engineering, Materials science

## Abstract

When grasping objects, humans actively adjust grip force in response to surface slipperiness and motion. Previous studies have showed that corrective actions occur after tactile afferents signal surface friction or slip events. However, the influence of the mechanical behavior of the skin on the development of contact forces is poorly understood. In this study, using contact kinematics derived from a natural reach-and-grasp task, we applied a glass surface onto restrained fingers via a robotic manipulator under low- and high-friction conditions. Contact forces were measured with a force sensor, and skin deformations were captured using a high-speed camera. As expected, the normal force remained unaffected by friction, however, interestingly the tangential force rose more slowly and peaked lower under low friction. This resulted in a higher normal-to-tangential force ratio, resembling friction-dependent scaling of grip-to-load force ratio observed in active grasping. The skin partially slipped throughout contact development, with the proportion of the slipped area first decreasing and then increasing. The time course of tangential force correlated with the extent of skin slip, both varying with friction. The findings demonstrate that skin mechanics potentially influences the grip stabilization during the initial phase of object handling, which doesn’t involve feedback-driven grip force adjustments.

**Supplementary Information:**

The online version contains supplementary material available at 10.1038/s41598-026-41781-z.

## Introduction

Human hands have evolved for dexterous object grasping and manipulation. They enable the stable grasp of objects varying in weight, shape, and slipperiness, relying on a versatile sensorimotor network. Sensory nerves innervating the skin, muscles, and tendons convey information about hand posture and interactions with objects. In parallel, the motor system plans and adjusts movements using this sensory feedback. Tactile cues about surface contact and properties are especially critical for a stable grip^1–4^.

Tactile afferents in glabrous skin respond to local deformation during contact^5–7^. They are influenced by the force and friction at the skin-surface interface^8–10^, which in turn, informs grip adjustments. To prevent slippage, the grip force (*F*_*G*_) must satisfy *F*_*L*_ *< µF*_*G*_ where *µ* is the coefficient of friction and *F*_*L*_ is the load force. If the grip-to-load force ratio is less than the reciprocal coefficient of static friction, called the slip ratio (i.e., *F*_*G*_*/F*_*L*_ < 1/*µ*), slippage occurs.

Major adjustments to grip force have been shown to occur in response to slip events detected by tactile afferents^3,7,11^. Nevertheless, such grip force adjustment can occur even without an obvious slip^12^. For example, humans can perceive surface slipperiness through submillimeter-range displacements at the skin-object interface^13,14^. However, processing tactile information takes time before it results in a motor response. Cortical responses to passive touch begin around 30ms after contact^15,16^, and corrective actions for the slip begin around 80ms post slip^2,3^, indicating initial grip control is feedforward. During this early pre-feedback phase, contact forces arise from the mechanical interaction between the skin and the object, governed by skin biomechanics, surface properties and imposed kinematics rather than sensory feedback.

During passive touch, the same principles apply. Even when objects contact the skin without motor involvement, the relationship between the normal and tangential forces in passive touch is closely analogous to the grip and load forces in active touch. It has been shown that when surfaces with varying friction were pushed against the skin in the absence of motor control, the measured tangential force was lower for surfaces with low friction. This difference appeared even though the evolution of the normal force was similar for high and low friction surfaces^14,17^. This results in an increased normal-to-tangential force ratio under low friction and resembles the behavior of grip-to-load force ratio changes under active motor control, which are primarily driven by active regulation of grip force. Since motor control is absent in passive touch, the normal and tangential forces are not strictly interchangeable with grip and load forces. However, skin mechanics influence the contact forces similarly during active and passive touch. Therefore, mechanisms observed in passive touch are partly involved in active grasping and may contribute to friction-related grip adjustments, especially during early pre-feedback phase of grip formation.

However, passive touch experiments typically isolate normal and tangential displacements, unlike natural movements where both interact and occur simultaneously. This limits our understanding of how friction and contact kinematics interact to shape early contact forces and tactile cues. To address this, we applied a glass surface on restrained fingers using a robotic manipulator reproducing the grip kinematics observed in a reach-and-grip task. We investigated the resulting contact forces, skin deformations and their interplay under varying friction conditions.

## Methods

### Ethical approval

Experiments were approved by Human Research Ethics Committee (approval number HC230352) at UNSW Sydney. The study conformed to the standards set by the Declaration of Helsinki, except for registration in a database. Participants read and signed the informed consent form after the experimental protocol was explained to them.

### Participants

Twelve participants (Group A, female/male: 3/9, 21–52 years old) performed a reach-and-grip task to record finger contact kinematics. The recorded contact kinematics were tested on another twelve (Group B, female/male: 6/6, 20–38 years old) in a passive touch protocol. Participants reported no history of finger injuries or conditions affecting finger pad mechanics. Before testing, they washed their hands and dried, then used alcohol wipes to remove residue.

### Mechanical stimuli

Group A participants were seated comfortably with their right arm aligned to easily reach a 260-gram object. When instructed, they freely reached, gripped, and lifted the object using the index finger and thumb (Supp. Video 1). The object and the hand were recorded in transverse plane at 100 frames per second with a high-definition camera (α6100; Sony, Japan; 1920 × 1080 pixels). Each participant completed 80 trials. A custom MATLAB code (2023b; The MathWorks, MA, USA) tracked the coordinates of markers on nails and object along grip (Z, dorsal-volar) and lift (Y, radial-ulnar) axes. Because the video recordings were aligned with the grip and lift axes, proximodistal movements could not be analyzed and were therefore ignored. Markers on each frame were re-referenced to the index finger marker, capturing contact kinematics from the finger’s perspective (i.e., as if the object was approaching the finger). Kinematics from the initial finger-object contact to lift-off moment were extracted and applied to the restrained fingers of Group B participants. No further analysis was carried out of data collected from Group A participants.

We analyzed 625 trials, excluding those with abrupt fluctuations, repeated contacts, and downward finger slippage. Contact kinematics were normalized so that the first contact coordinate was the origin (y = 0, z = 0), and lift-off was (y = 1, z = 1). Data from all participants was pooled and clustered to identify common kinematic patterns in MATLAB. First, pairwise similarities on Y and Z axes were estimated between each kinematics using dynamic time warping, then averaged geometrically. The resulting similarity matrix was reduced to two dimensions using non-metric multidimensional scaling and clustered using k-medoids, maximizing the Silhouette score. k-medoids was chosen due to its robustness to outliers and compatibility with custom distance metrics^18^. Clusters with fewer than three samples or with high acceleration beyond the safety limits of experimental equipment were excluded. This yielded 17 clusters which were fitted with Eqs. (1) and (2). Equation (1) defines the position of surface along the dorsal-volar axis (*z(t)*; normal to the skin) as a function of time (*t*), and Eq. (2) defines the position of the surface along the radial-ulnar axis (*y(t*,* z)*; tangential to the skin) as a function of both time and *z(t)*. This set of equations captured non-linear kinematics well (R^2^ ≥ 0.85) (Supp. Fig. 1).

1 $$z\left(t\right)=k{e}^{lt}+m{e}^{nt}$$

2 $$y\left(t,z\right)=\frac{z\left(t\right)}{\left(1+{e}^{\raisebox{1ex}{$\left(a-t\right)$}\!\left/\!\raisebox{-1ex}{$b$}\right.}\right)}$$

where *e* is the Euler’s number, and [*a*,* b*,* k*,* l*,* m*,* n*] are the constants to be fit to data.

Generalized kinematics were regenerated using Eqs. (1) and (2) with a 2 mm peak displacement in the radial-to-ulnar direction and a dorsal-volar displacement calibrated to produce 1.2 N indentation force on the finger pad. The 2-mm displacement in the radial-to-ulnar direction was based on video analysis showing average finger pad displacement relative to the nail marker from contact to lift-off. Dorsal-volar displacement was individually calculated for Group B participants using pre-test indentation-force calibration data.

**Fig. 1:**
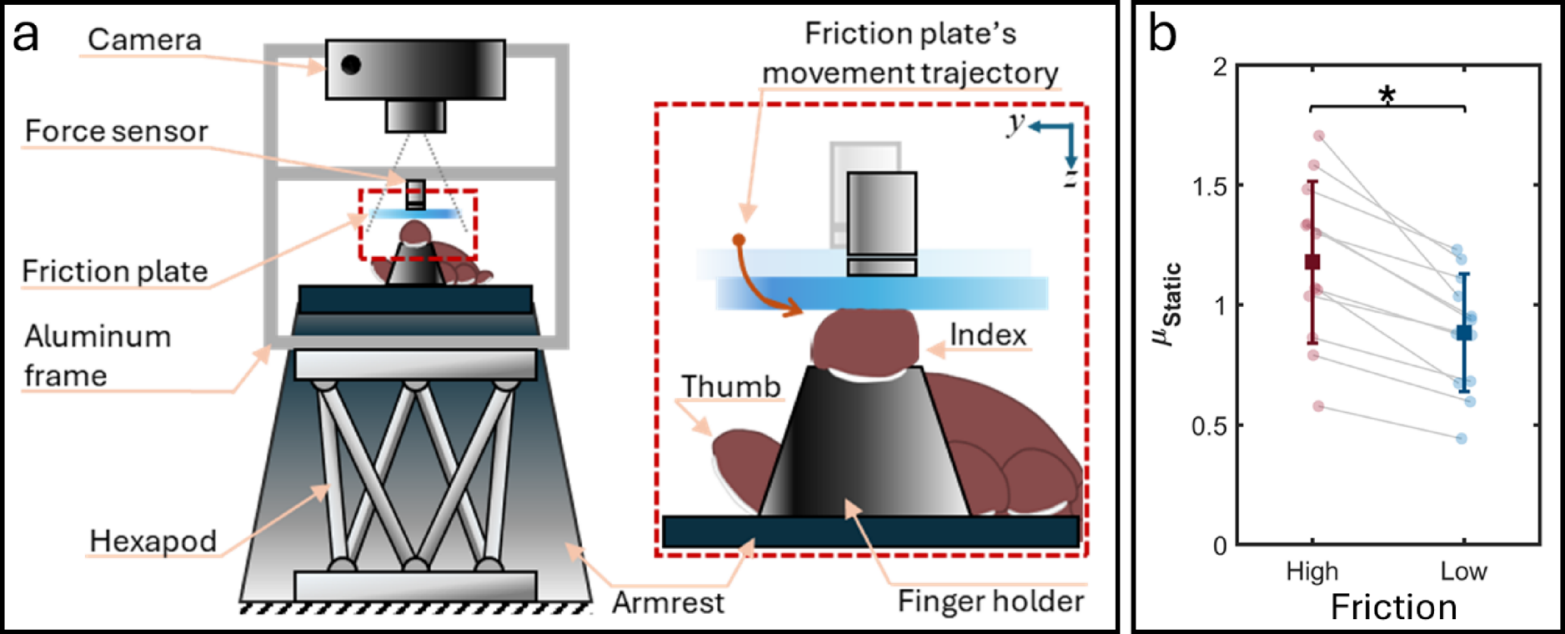
Experimental setup and surface frictions. (**a**) The friction plate was mounted on an aluminum frame through a torque/force sensor and carried by the hexapod based on the contact kinematics derived from the reach-and-grip task. The index finger was restrained in a finger holder under the friction plate. To minimize involuntary finger movements, participant’s forearm was stabilized using a suction pillow on an armrest. (**b**) Coefficient of static friction (*µ*_*S*_) was significantly different between low- and high-friction surfaces (*p < 0.001). Dots paired with gray lines show the *µ*_*S*_ measured from the same participant. Squares show the mean and error bars show one standard deviation.

A robotic manipulator was driven using these kinematics to stimulate the finger pad with the smooth surface of a friction reduction device (Fig. 1a). Each kinematics was tested with six repetitions under low- and high-friction conditions in pseudorandom order, with low- and high-friction conditions being delivered consecutively to observe immediate differences between the two under consistent skin conditions.

### Friction reduction device

Details of the ultrasonic friction reduction device have been reported by Wiertlewski, et al.^19^ and in prior work^14,17^. Briefly, the clear and smooth glass surface of an ultrasonic friction reduction device (friction plate) was used as the stimulation surface. When actuated at 33 kHz, piezoelectric elements (SMPL131W89T10; Steminc, FL, USA) on the glass generated ultrasonic vibrations that reduced the friction via slight levitation of the skin above the glass. Friction was controlled by adjusting vibration amplitude. The signal used to drive piezoelectric elements was produced by a function generator (DG 1022; RIGOL Technologies, China) and amplified using a high-voltage amplifier (A-303; AA Lab Systems Ltd., Israel). A custom MATLAB script, referred to as *the control program*, controlled the output amplitude of the function generator via a data acquisition card (USB-6218; National Instruments, TX, USA). We tested two friction conditions: high friction (device off) and low friction (device at 90% power).

### Robotic manipulator

The friction plate was mounted on an aluminum frame via a force/torque sensor (Nano17; ATI Industrial Automation, NC, USA) and carried by a 6-axis robotic manipulator (H-840 Hexapod; Physik Instrumente, Germany) (Fig. 1a). The MATLAB script generated and sent the contact kinematics to the robotic manipulator’s controller (C-887 Hexapod Controller; Physik Instrumente). The manipulator executed each trajectory with a 1 μm precision at 10 kHz. Contact forces at the interface of friction plate and finger pad were recorded via the force sensor using two data acquisition devices for stimulus control in MATLAB (USB-6002; National Instruments, TX, USA) and for data analysis in LabChart (PowerLab 16/35; AD Instruments, New Zealand). The control program generated a synchronization signal via an analog output of USB-6002 to be recorded by LabChart and video camera (see below). Before each trial, the control program positioned the friction plate ~ 0.5 mm above the skin by first indenting the skin to ~ 0.1 N, while recording the position and force, then retracting the friction plate based on the position-force curve.

### Friction measurement

We measured the coefficient of static friction (*µ*_*S*_) under low- and high-friction conditions at the start and end of the experiment, with six repetitions per condition. The robotic manipulator applied a 1.2 N normal force, then moved the friction plate 10 mm in the ulnar direction at 5 mm/s to induce slip. *µ*_*S*_ was calculated using the recorded normal and tangential forces at slip onset, identified by a clearly defined peak in tangential force indicating that the maximum load-bearing capacity of the skin had been reached and slip occured^17^. Mean *µ*_*S*_ (± standard deviation) was 0.88 ± 0.24 under the low-friction condition and 1.18 ± 0.34 under the high-friction condition (paired t-test, *t*(11) = 6.48, *p* < 0.001) (Fig. 1b).

### Video recordings and image processing

Video recording and image processing methods were previously described^13,17^. Briefly, a camera (α6100; Sony, Japan), mounted above the friction plate, recorded finger pad through the clear glass of the friction plate (1920 × 1080 pixels; 100 fps; field of interest: 65 mm×40 mm). High-contrast imaging of fingerprint ridges was achieved using total internal reflection principle. The synchronization signal generated by the control program was recorded by the camera’s sound input.

Fingerprint images from perspective-corrected frames were extracted using Otsu’s thresholding. Features were detected with the minimum eigenvalue algorithm^20^ and tracked using the Kanade-Lucas-Tomasi algorithm^21^. Tracking started at the frame of the peak normal force and ended when fewer than five features remained or the kinematic execution finished. Features not tracked for five consecutive frames were excluded.

Delaunay triangles were formed from tracked features. The movement vectors were constructed for triangle centers at incremental levels of the normal force (5%, then 10–100% in 10% increments). Each pixel within a triangle was assigned with the displacement vector of that triangle. Slip was defined as displacement exceeding one pixel. Net displacement and slip percentage within contact area were estimated across force increments.

### Statistical analysis

We assessed data distribution using the Kolmogorov-Smirnov test. Paired-sample *t*-test was used for normally distributed variables, and Wilcoxon signed rank test for non-normal data. Holm-Bonferroni adjusted p-values were reported for multiple comparisons. Linear mixed-effects models (LMM) were fitted using restricted maximum likelihood to analyze the effects of friction condition, contact kinematics, and covariate relationships on contact forces and properties (e.g., contact area, slips). The models included main effects and pairwise interactions, and random effects due to subject variability. Modelling was followed by analysis of variance with Satterthwaite approximation to adjust degrees of freedom, and both F-values and p-values are reported. All analyses were performed in MATLAB.

## Results

### Effects of friction on contact forces

**Fig. 2:**
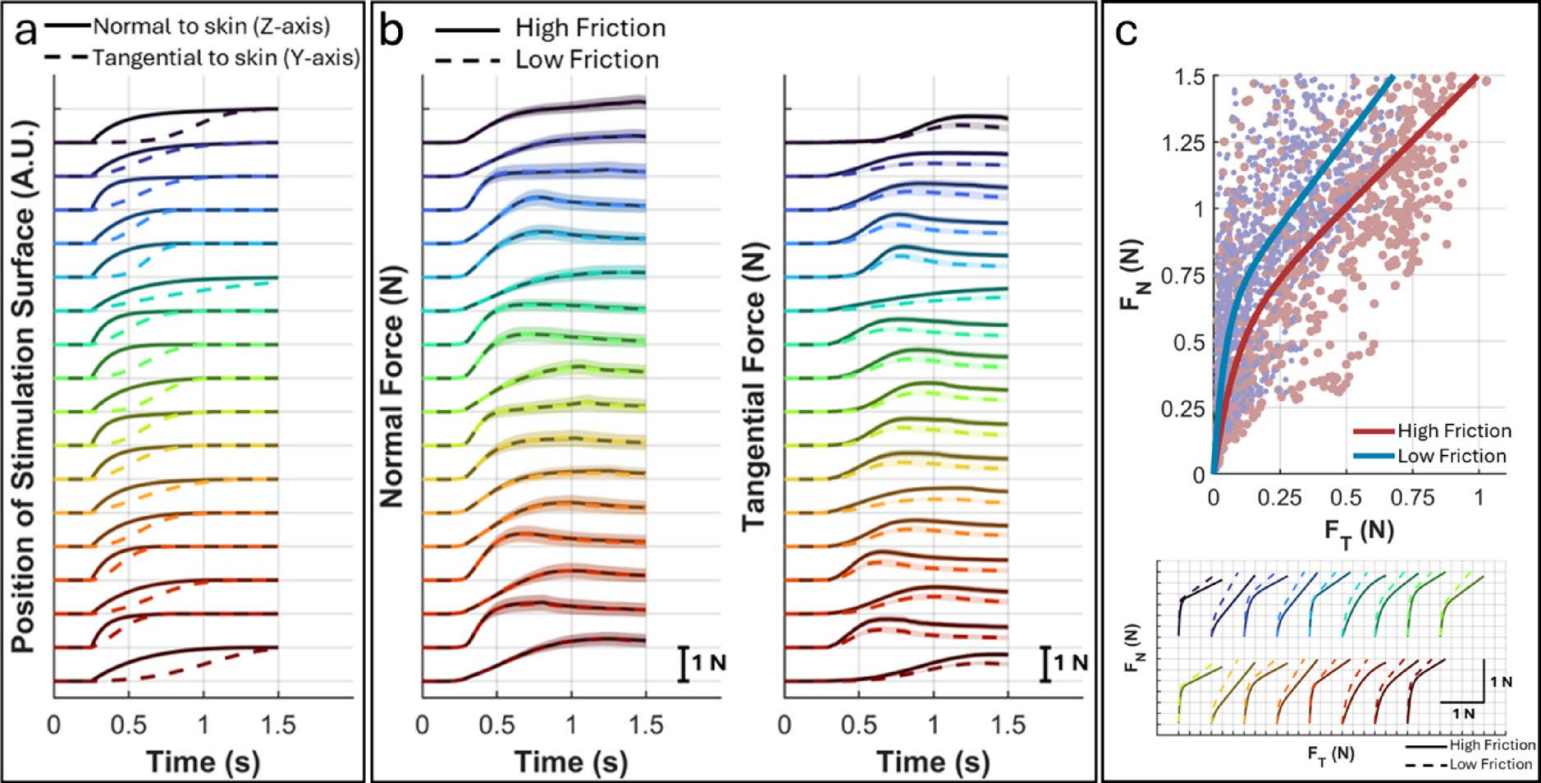
Contact kinematics and friction significantly affected the development of contact forces. (**a**) Contact kinematics applied on the restrained fingertips. (**b**) Normal force development differed between kinematics but was not affected by friction, while tangential force was influenced by both kinematics and friction. (**c**) Relationship between normal and tangential forces was depending on friction and contact kinematics. For each participant, normal and tangential force recordings were averaged across repetitions and plotted as individual data points to illustrate the variability during contact development up to the peak tangential force. Solid lines show the average across all trajectories and participants. Contact kinematics and related contact forces are color coded and matched between panels. Shaded areas show 95% confidence interval. A.U.: arbitrary units.

Figure 2 shows the tested kinematics and the resulting normal and tangential forces averaged across participants of Group B. Inherently, contact force dynamics, such as rate of change and relative timing between tangential and normal forces, were correlated with kinematics (Spearman’s rho > 0.6, *p* < 0.001). For example, the position of the stimulation surface and the force along the same axis were correlated.

We first analyzed the effects of friction condition and contact kinematics on contact forces using LMM. Peak normal force was similar between friction conditions (1.31 ± 0.38 N under low friction and 1.32 ± 0.38 N under high friction, F(1,42.1) = 0.339, *p* = 0.564) and unaffected by kinematics (F(16,192) = 1.22, *p* = 0.255). It should be noted that although a pre-test calibration was performed to determine the skin indentation required to achieve a normal force of ~ 1.2 N, the execution of the contact kinematics was position-controlled. As a result, the normal force was expected to deviate from 1.2 N at the end of stimulus due to finger pad bulging induced by lateral movement. The peak rate of change of normal force also showed no differences (4.29 ± 2.08 N/s under low friction and 4.27 ± 2.04 N/s under high friction, F(1,203.3) = 0.024, *p* = 0.876; kinematics: F(16,192) = 0.971, *p* = 0.490).

Tangential force increased at a slower rate under low friction (1.76 ± 0.76 N/s; F(1,24.8) = 18.5, *p* < 0.001) and plateaued at a lower peak (0.55 ± 0.16 N; F(1,17.7) = 38.8, *p* < 0.001) compared to high friction condition (rate: 2.44 ± 0.92 N/s, and peak: 0.83 ± 0.16 N). Kinematics significantly affected both peak tangential force and its peak rate of change (peak: F(16,192) = 4.45, *p* < 0.001; rate: F(16,192) = 3.84, *p* < 0.001). Plotting normal force against tangential force revealed a friction-scaled relationship (Fig. 2c), consistent with behavioral studies where grip-load forces coupling is scaled by friction^2,3^.

### Friction-dependent time course of tangential force

Because the friction affected the tangential force development, we measured the time it took for tangential force to reach 0.1 N after normal force reached 0.1 N, which is referred to as tangential force (F_T_) delay hereafter. We selected 0.1 N as the reference point based on our previous studies, which demonstrated that reliable friction discrimination is possible above this level^14,17^, indicating a sufficient number of tactile events for performing the task. Nonetheless, it does not represent a perceptual threshold or imply tangential force is the primary cue for friction perception but used as a reference point to explore possible early friction-dependent mechanisms contributing to grip control.

**Fig. 3:**
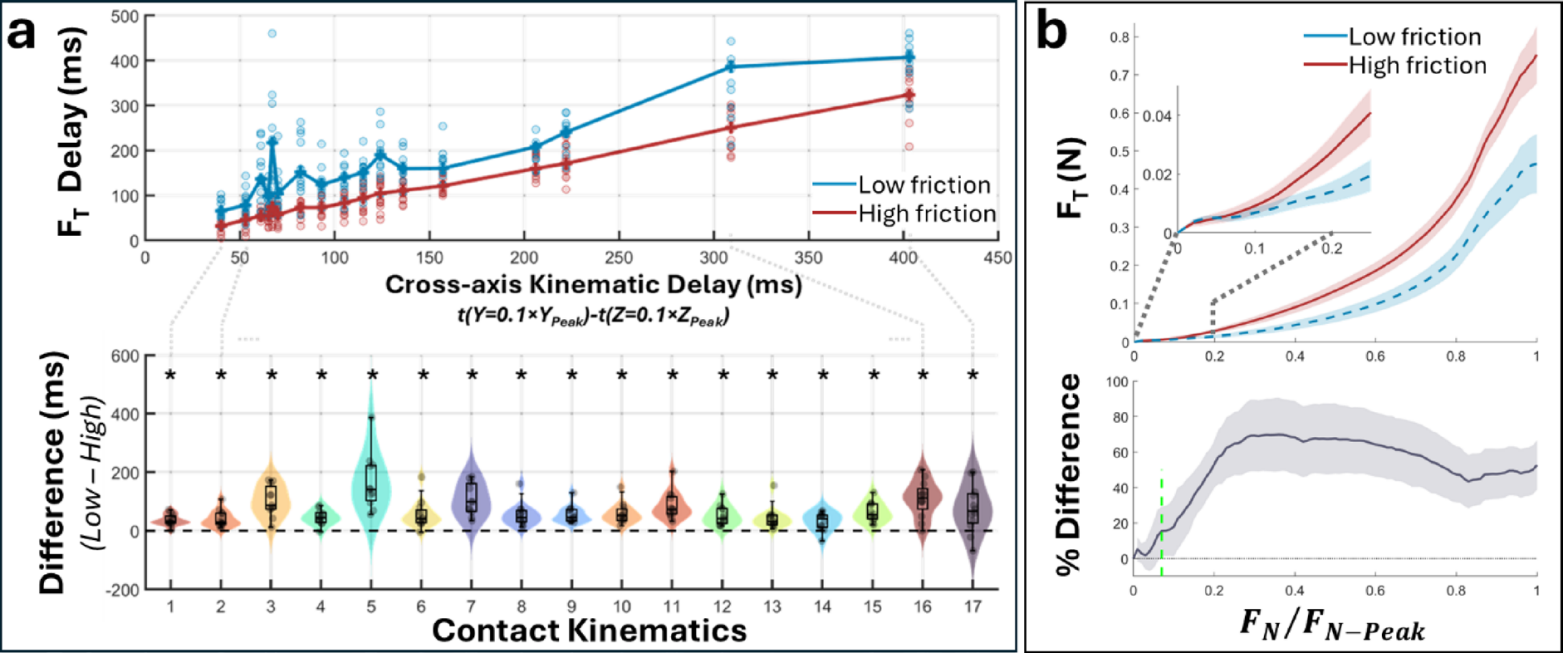
Tangential force (F_T_) increased at a lower rate under low-friction condition compared to high-friction condition. (**a**) F_T_ delay was defined as the time it took for F_T_ to reach 0.1 N after F_N_ reached 0.1 N. It correlated with the cross-axis kinematic delay (top graph). The difference between low- and high-friction conditions was always significant and relatively similar across all contact kinematics. Circles show the mean values of individual participants. Asterisks indicate the difference between friction conditions was greater than zero (*p* < 0.038). (**b**) On average across all tested kinematics, tangential force was generally smaller under low-friction condition during contact development. The 95% confidence interval for the difference between friction conditions (grey shaded area) excluded zero once the normal force exceeded ~ 7% of its peak value (green dashed line).

Using an LMM, we analyzed how friction condition and contact kinematics influenced tangential force delay. F_T_ delay was longer under low friction (174 ± 96ms) than under high friction (108 ± 80ms; F(1,98.7) = 12.0, *p* < 0.001) and was also affected by kinematics (F(16,87.1) = 22.7, *p* < 0.001) (Fig. 3a and Supp. Fig.  2). It increased with increasing cross-axis kinematic delay, defined as the time between normal displacement reaching 0.1×z_peak_ and tangential displacement reaching 0.1×y_peak_. Nevertheless, the effect of friction was similar and significant across all kinematics (Wilcoxon test: all z<-2.43, all *p* < 0.038).

We further analyzed the tangential force at incremental normal force levels (1-100% of peak with 1% steps). For a given normal force level, tangential force was usually higher under high-friction condition (Fig. 3b). This was mainly due to the reduced rate of change of tangential force under low-friction condition. The tangential force began to differentiate between friction conditions early on, as the 95% confidence interval for their difference excluded zero once the normal force exceeded ~ 7% of its peak value.

### Friction-dependent evolution of slip behavior

We analyzed how friction condition and contact kinematics influenced contact properties (e.g., contact area, percentage of slipped area, and net cumulative slipped distance) across incremental normal force levels (5%, then 10–100% of peak with 10% steps) using LMM. Development of contact area varied significantly with kinematics (F(16,4221.9) = 9.88, *p* < 0.001), and friction (F(1,4221.8) = 4.53, *p* = 0.033). There was also a significant interaction between kinematics and friction (F(16,4221.9) = 1.94, *p* = 0.013) (Supp. Fig. 3). However, peak contact area was similar between conditions (kinematics: F(16,396) = 0.504, *p* = 0.945; friction: F(1,396) = 0.592, *p* = 0.44; interaction: F(16,396) = 0.528, *p* = 0.932).

**Fig. 4:**
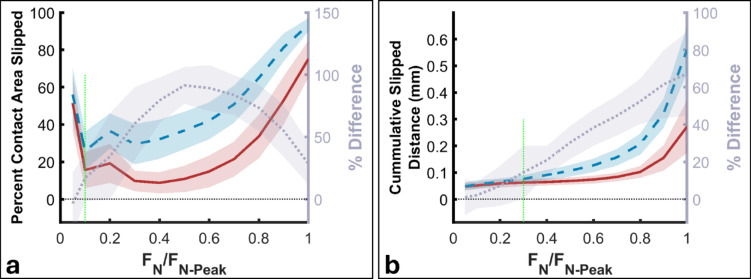
Slipping contact area changed during contact development. (**a**) The slipped area initially decreased, indicating that more skin adhered to the surface as normal force increased, but later began to rise, likely due to growing tangential displacement. Slipped area was consistently larger under low-friction conditions, with the difference between friction levels becoming evident once normal force exceeded 10% of its peak value (green dashed line). (**b**) Slipped distance increased throughout contact development, with the difference between friction conditions emerging as normal force exceeded 30% of its peak (green dashed line). Vertical green dashed lines indicate the normal force at which the 95% confidence interval for the difference between low- and high-friction conditions excluded zero. Shaded regions represent the 95% confidence interval.

Slipped contact area differed between friction conditions (Fig. 4a and Supp. Figs.  4 and 5a), with a larger proportion of contact area slipped under low friction (F(1,4221.9) = 9.25, *p* = 0.002), as the 95% confidence interval for the difference between friction conditions excluded zero once the normal force exceeded 10% of its peak value. Kinematics also affected slipped area (F(16,4222) = 10.2, *p* < 0.001) and interacted with friction (F(16,4222) = 12.1, *p* < 0.001). To explore early friction-kinematics interactions, we analyzed correlation between slipped area at F_T_=0.1 N and cross-axis kinematic delay. The correlation was stronger under low friction (Spearman’s rho=-0.460, *p* < 0.001) than under high friction (rho = -0.173, *p* = 0.016) (Supp. Fig.  5b). As the cross-axis kinematic delay increased (i.e., the surface had already moved further along the normal axis before tangential displacement began), slipped contact area decreased. This is likely due to greater normal force and contact area before tangential movement, enhancing friction and skin adhesion to the surface.

Net cumulative slip distance was greater under low friction (F(1,4221.3) = 13.1, *p* < 0.001) (Fig. 4b), as the 95% confidence interval for the difference between friction conditions excluded zero once the normal force exceeded 30% of its peak. Kinematics significantly affected slip distance (F(16,4221.4) = 3.73, *p* < 0.001) and interacted with friction (F(16,4221.3) = 6.03, *p* < 0.001) (Supp. Fig. 6). Under low friction, slip distance was correlated with cross-axis kinematic delay (Spearman’s rho=-0.400, *p* < 0.001), but not under high friction (rho = -0.126, *p* = 0.083).

### Relation of tangential force with slip behavior

**Fig. 5:**
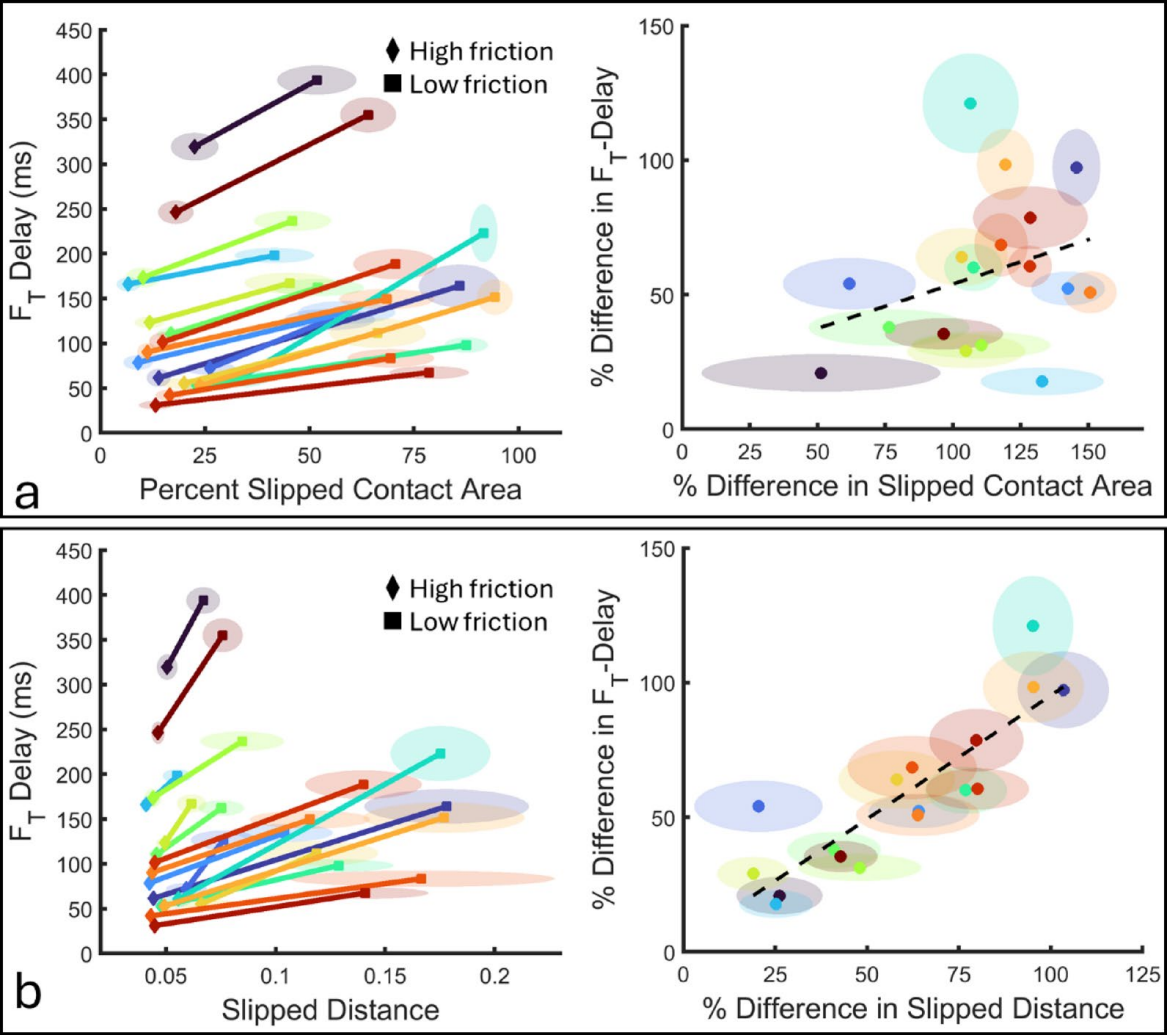
Changes in contact properties were associated with changes in F_T_ delay across friction conditions. Slipped contact area (**a**) and slip distance (**b**) differed between friction conditions (left panels). The magnitude of these changes with friction was significantly associated with the change of F_T_ delay between friction conditions (right panels). Centroids show the mean and shaded area shows the 95% confidence interval. Different colors represent different kinematics.

We used LMMs to test whether changes in slipped contact area and cumulative slip distance with varying friction were associated with tangential force and its delay across friction conditions. The tangential force delay was affected by friction-dependent changes in slipped contact area (F(1,178.5) = 10.7, *p* = 0.001) and net cumulative slip distance (F(1,173.4) = 40.0, *p* < 0.001); tangential force was *delayed* more with increasing slipped area and cumulative slip distance measured at F_T_=0.1 N (Fig. 5).

**Fig. 6:**
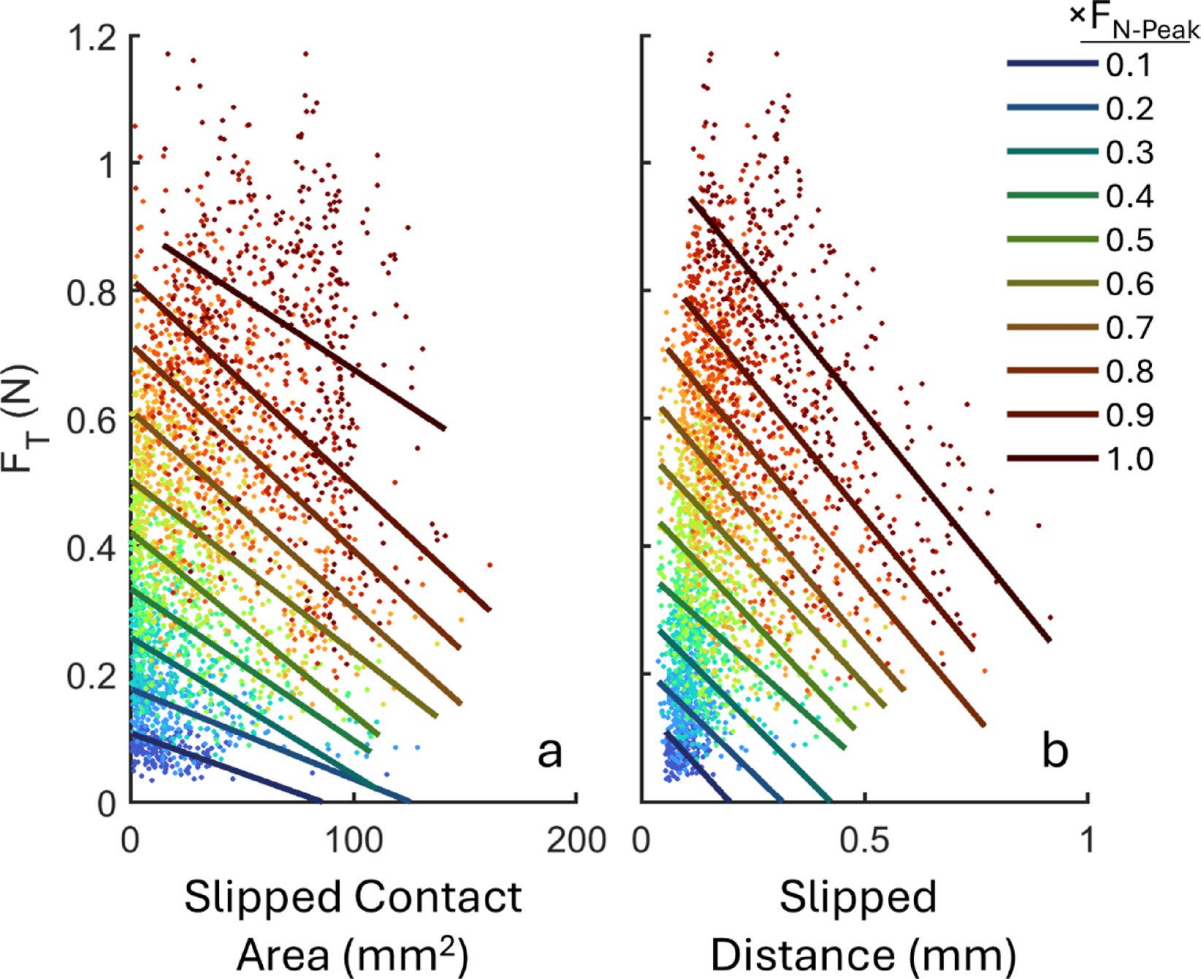
Tangential force decreased with (**a**) increasing slipped contact area and (**b**) cumulative slip distance during contact development (F_N_: 0.1–1 N in 0.1 N increments). Data from all contact kinematics and friction conditions were pooled together across subjects and color-coded by F_N_ level. Each point represents the average of repetitions under a given condition. Solid lines are the first order polynomial fits for each F_N_ level.

Tangential force was also associated with both slipped area (F(1,2672) = 8.48, *p* = 0.004) and net cumulative slip distance (F(1,2656) = 12.4, *p* < 0.001); it decreased with increasing slipped area and slip distance at all levels of normal force (Fig. 6).

## Discussion

Previous studies have generally focused either on active adjustments to grip force in response to changes in friction and load, or on measuring frictional responses using independently controlled movements normal and tangential to the skin. In this study, we used contact kinematics from behavioral experiments to investigate how skin deformation and contact forces are influenced by kinematics and friction. Our findings revealed how friction- and kinematic-dependent mechanisms influence the development of contact forces, particularly tangential force during the initial phases of grip before sensory-feedback driven grip force adjustments are made. These results suggest that skin mechanics and contact kinematics play a critical role in shaping grip force development from the onset of object interaction.

Pre-lift contact kinematics tested in this study involved simultaneous normal and tangential movements with the relative timing differing between different kinematics. That allowed us to observe behavior of the skin during the initial phases of the contact. We found that partial skin slip occurs early in contact and is modulated by both friction and kinematics. Lower friction resulted in a greater slipped skin area and slip distance, consistent with our previous behavioral findings^13^, with the effect size influenced by kinematic dynamics.

Slip occurs when tangential force acting on a contact interface exceeds friction force, which depends on normal force and the coefficient of friction. Because pressure is not evenly distributed across the contact area due to the morphology of the finger pad, local friction as well as shear force varies leading to partial slips. Under low friction, less skin area could adhere to the contacting surface yielding an increase in partial slip immediately upon contact compared to high friction. However, the slipped area decreased briefly after contact, suggesting an increase in adhesion as contact developed, and then increased again. This phenomenon was probably a result of skin viscosity and changes in friction over the course of contact development due to increasing normal force and lateral displacement. Although the friction coefficient can increase with lateral traction speed of kinematics^22,23^, this may not offset the skin’s viscous damping (i.e., resistance to movement), leading to greater slippage. Nonetheless, over the initial course of contact kinematics, interaction between surface asperities increases causing more skin to stick on surface. Subsequently, as normal force increases, the friction coefficient decreases because skin asperities flatten reducing interactions between surfaces^23–30^. However, overall friction force still increases because normal force and contact area grow faster than the coefficient drops. This, in return, causes the tangential force to increase with lateral traction until it again exceeds friction force, resulting in slip.

We observed that both the rate of increase and peak tangential force decreased under low friction, while the normal force remained unaffected. This produced a friction-dependent increase in the normal-to-tangential force ratio, consistent with behavioral studies where grip-to-load forces ratio is scaled by friction. However, in active touch, grip force is adjusted, after a certain delay, by motor system based on the sensory inputs from different modalities and can significantly change based on the friction, while the load force depends on the object being lifted^3,11,31,32^. On the other hand, in our experiments, normal force (analogous to grip force) did not vary with friction, but tangential force (analogous to load force) did. Nevertheless, passive friction-dependent coupling between contact forces is unavoidable in active object gripping due to the nature of contact kinematics and the mechanical behavior of the skin.

We found that friction-dependent variation in tangential force development was associated with different slip-stick patterns between friction conditions; larger slipped area and greater slip distances under low friction reduced tangential force. When friction conditions were pooled together, tangential force was inversely correlated with slipped area and slip distance, as the skin releases shear stress through slips.

Critically, skin did not fully stick to the surface immediately after the contact, but instead it partially slipped until a friction force that is enough for skin to stick to the surface was developed. Slip-stick events during this phase determined the increase of tangential force, explaining the distinct patterns between friction conditions. This finding aligns with prior work showing that finger pad expands radially on the surface during grip^8^ and that tangential force decreases under lower friction^14,17^. For example, Afzal et al.^14^ applied lateral displacements as small as 0.2 mm after lowering a glass surface on fingertip and observed reduced tangential force under lower friction. Although slip events were not analyzed in this study, participants’ ability to discriminate between surface frictions suggest that local slips occurred, providing sufficient tactile cues for the task.

In this study, partial slips generally occurred from the very beginning of contact, and some parts kept slipping through the course of contact kinematics. Therefore, we didn’t control our analysis for either dynamic or static friction acting at different parts of the contact area. It was necessary for contact forces to exceed a certain slip ratio to initiate a slippage (even at the onset of the contact), but we didn’t measure the coefficient of static friction at such low contact forces. Previous studies have shown that the skin’s coefficient of friction decreased with increasing normal force^23–30^. Accordingly, the coefficient of friction may have decreased over the course of contact development as normal force increased, although the rate of decrease may differ between friction conditions. In the literature, some methods for continuous friction measurement have been proposed^26^, which rely on continuous slippage. However, these methods are not applicable in our case, as we did not consistently observe full slips but only local slip-and-stick events during early contact development.

The contact kinematics tested in this study were collected from participants who grasped and lifted a 260-gram object. The weight of the object may influence contact kinematics and, consequently, contact development, as grip and load forces and their rates scale proportionally with object weight to ensure a safe grip before lifting. We would expect these effects to be particularly prominent during the later stages of contact development, whereas object weight would influence the initial contact development, and thus our observations at this stage, to a lesser degree. However, this needs to be investigated in future studies.

The contact kinematics tested in this study were extracted from the index finger during a precision grasp task. However, natural grasping encompasses a wide range of grasp types^33^ which vary in contact configuration, force distribution, and motor coordination, thereby influencing how contact forces develop under different friction conditions. In a previous study involving free reaching and active motor control to touch a surface, we observed similar patterns of early skin slip during contact^13^. The consistency of these observations across constrained and unconstrained contact scenarios suggests that the mechanisms identified here are representative of real-world precision grasping. Nevertheless, the extent to which these findings generalize to other grasp types involving additional digits, palm contact, or different kinematic strategies remains to be investigated.

We didn’t measure the mechanical properties of the skin such as stiffness and elasticity, which would have significant effects on development of contact forces. For example, softer skin might deform more, increasing the contact area, and thus, the friction force. In this study, we can only conclude that the same trend of effect of friction conditions is valid for each participant regardless of mechanical properties of their finger pads.

The literature suggests that it usually takes more than 80ms for motor system to adjust for grip force based on incoming sensory information^2,12,34^. Therefore, the early execution of the gripping movement is mostly under feedforward control that depends on expectations based on visual inputs and previous experiences^35–37^. Based on the present findings, we suggest that the passive mechanical behavior of the finger pads may partially contribute to the development of grip between the fingertip tissue and surface, especially in the early stages of contact. This passive mechanism and viscoelastic nature of the finger pads may improve the grip stability until a reflexive adjustment can be achieved, especially in dynamic and unpredictable environments. The damping effect of the finger pads might also be useful for absorbing and dissipating perturbational forces. However, our understanding of how mechanical structure and properties of the skin influences its behavior under different friction conditions is limited^38,39^ and this area warrants further investigation.

## Supplementary Information

Below is the link to the electronic supplementary material.

Supplementary Material 1

Supplementary Material 2

## Data Availability

The data generated and analyzed in the current study are available from the corresponding author on reasonable request.
