# Supplementary material for: The role of skin mechanics in contact force variation under different friction conditions
Source: Sci Rep. 2026 Mar 1;16:11481. doi: 10.1038/s41598-026-41781-z (PMC13057093; doi:10.1038/s41598-026-41781-z)
Supplement: Supplementary file 1 — Supplementary Material 1 [file 41598_2026_41781_MOESM1_ESM.pdf]

The role of skin mechanics in contact force variation under different friction conditions

Supplementary Video

A video is provided showing behavioral experiments performed with Group-A participants and passive touch experiments performed with Group-B participants. In behavioral experiments, participants freely reached and grasped a 260-g object using a precision grip. Contact kinematics from initial contact to object lift were extracted from video recordings. Contact kinematics of the index finger on grip and lift axes were then re-referenced such that the finger was stationary, and the surface moved relative to it. These contact trajectories were subsequently used to apply the glass surface of a friction modulation device to the restrained fingers of Group-B participants using a robotic manipulator. For privacy reasons, fingerprints visible in the passive touch videos were blurred.

Supplementary Figures

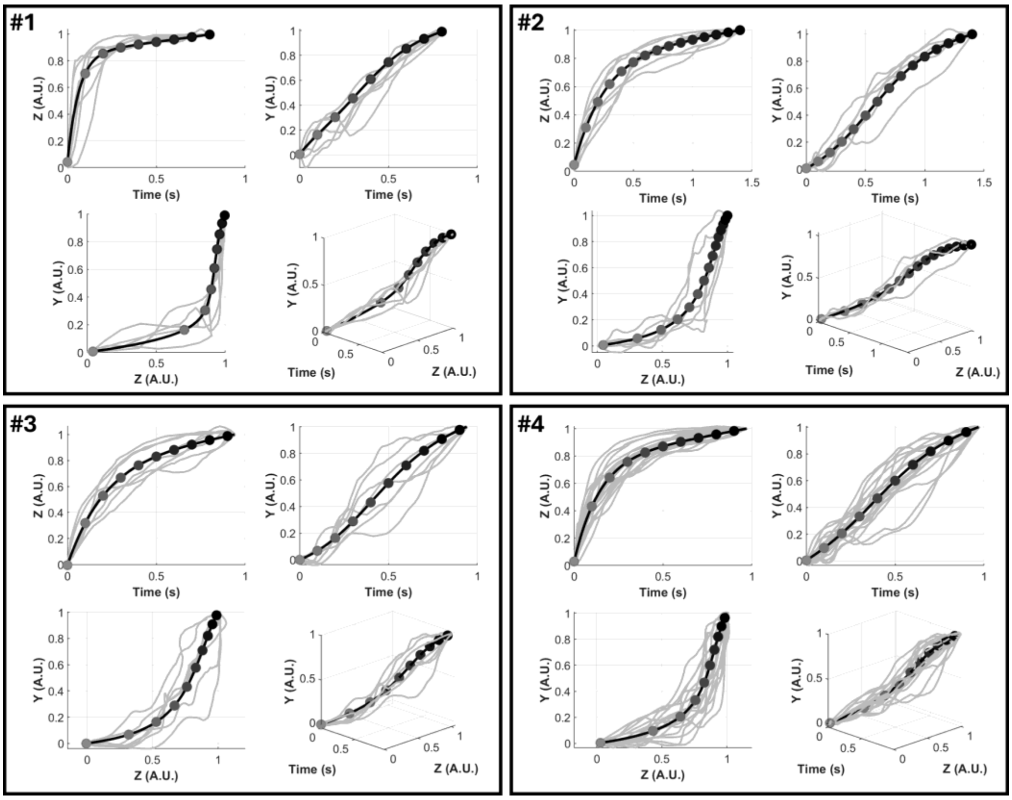

Supp Figure 1. Four exemplary contact trajectories (kinematics) tested in this study. Gray lines represent raw trajectories extracted from video recordings of reach-and-grasp task performed by Group A participants. Black lines represent curves fitted to the raw data. Circles indicate position at 100-ms intervals with darker colors corresponding to later time points along the trajectory. These markers are included to facilitate comparison across trajectories. Each panel shows displacement normal to the skin (Z) as a function of time (top left), displacement tangential to skin (Y) as a function of time (top right), the phase plot of Y vs Z (bottom left), and the two-dimensional trajectory over time (bottom right). Trajectories were normalized so that the first contact coordinate was the origin (y=0, z=0), and lift-off was (y=1, z=1). Data from all participants was pooled and clustered to identify common kinematic patterns as described in methods section. Time is in seconds. A.U.: arbitrary unit.

The role of skin mechanics in contact force variation under different friction conditions

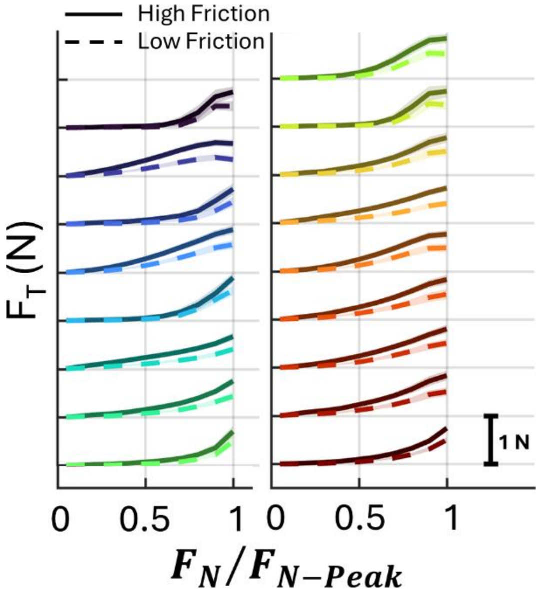

Supp Figure 2. Change of tangential force depended on both contact kinematics and friction. In each tested contact kinematics, tangential force increased at a higher rate under high-friction condition (solid lines) compared to the low-friction condition (dashed lines).

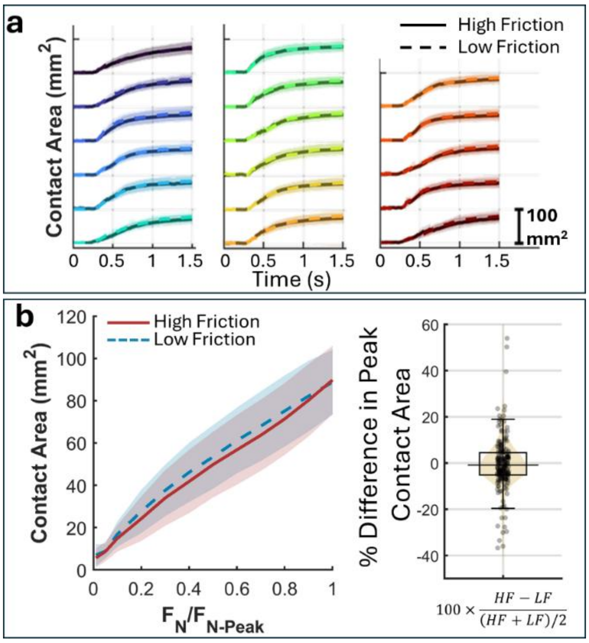

Supp. Figure 3. Contact area development depended on kinematics and friction, but the final value was similar across conditions. (a) Contact area increased similarly under both friction conditions but varied with kinematics. (b) Across different normal force levels, both factors influenced development (left panel). Final contact area remained similar across conditions (right panel). Shaded areas indicate the 95% confidence interval.

The role of skin mechanics in contact force variation under different friction conditions

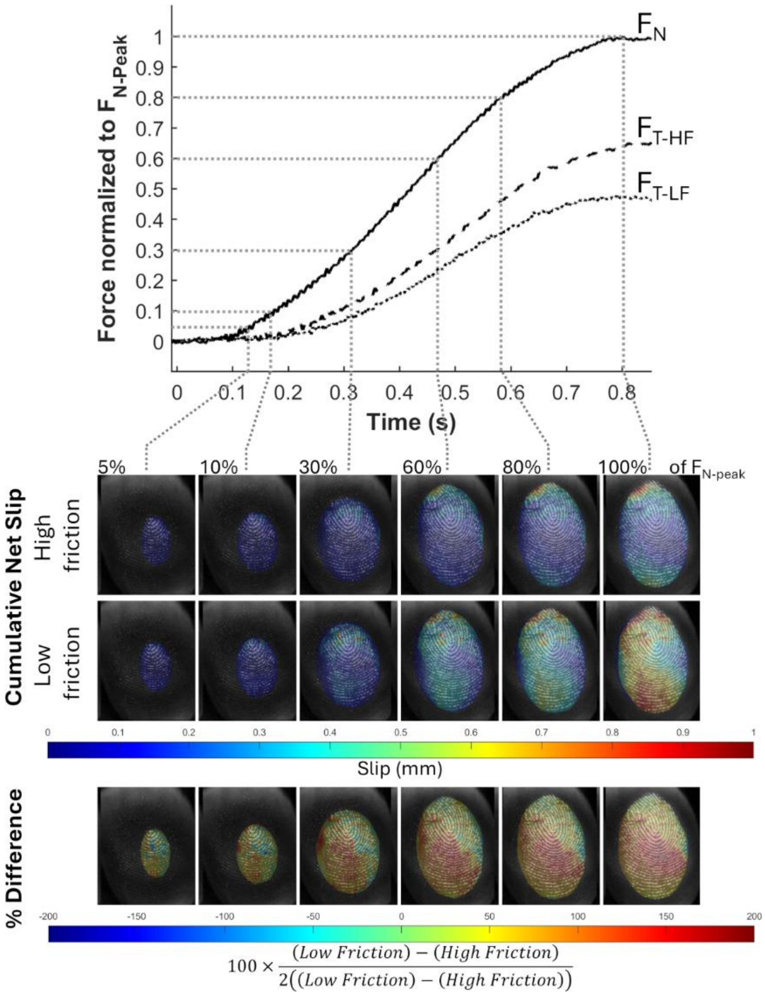

Supp Figure 4. An exemplar skin displacement analysis with normalized contact forces under different friction conditions. Normal force (F_N) was not affected by friction condition, but tangential force (F_T) did (top panel; LF: low friction, HF: high friction). The skin slipped more under low friction (middle panels) and the difference was apparent even at a very early stage of the contact (bottom panel).

The role of skin mechanics in contact force variation under different friction conditions

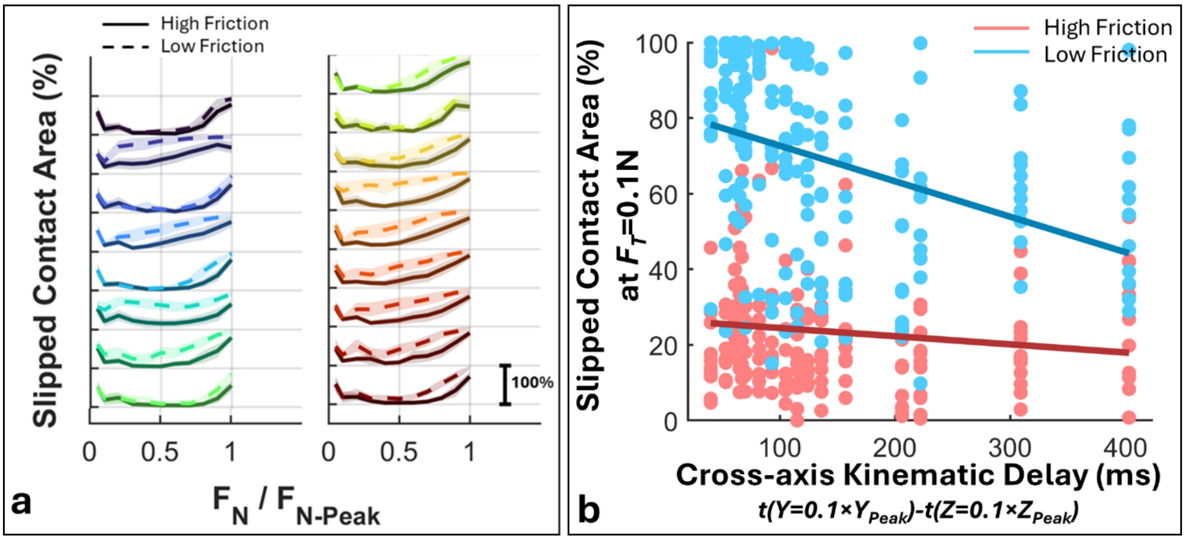

Supp Figure 5. The size of the slipped contact area changed during contact development depending on the contact kinematics. (a) The slipped area first decreased and then increased during contact development for each kinematics. (b) At F_T=0.1N, the slipped area decreased as the cross-axis kinematics delay increased; for example, when lateral displacement is slower than skin indentation, a smaller skin area slipped. Data points represent average of six repetitions per participant for each friction condition and kinematics. Solid lines indicate first-order trend lines.

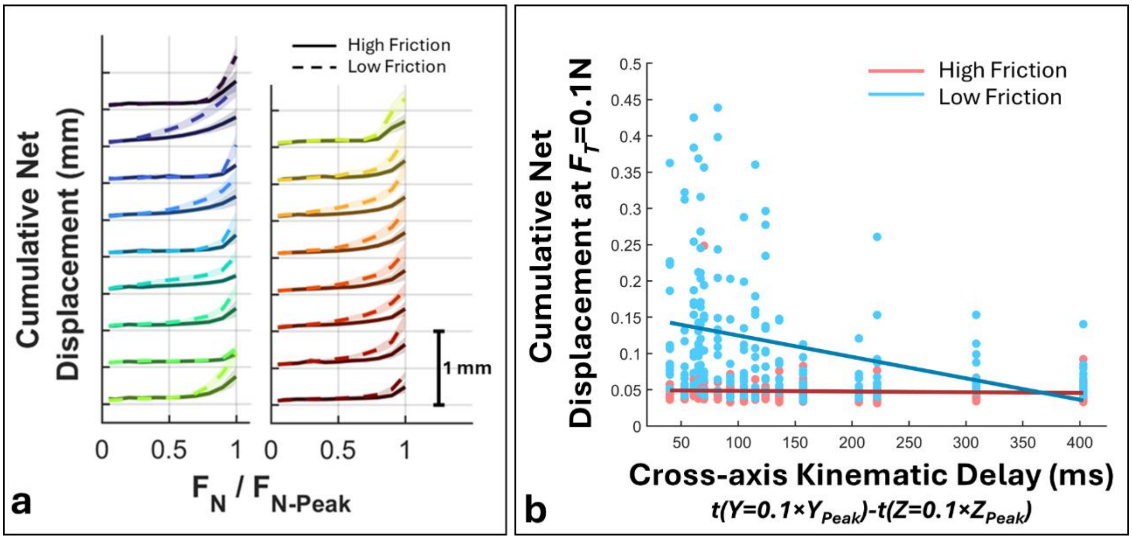

Supp Figure 6. Cumulative slipped distance increased during contact development. (a) The slipped distance was bigger under low friction condition compared to high friction for all kinematics. (b) At F_T=0.1N, the slipped distance decreased as the cross-axis kinematics delay increased; for example, when lateral displacement is slower than skin indentation, the skin slipped a shorter distance. Data points represent average of six repetitions per participant for each friction condition and kinematics. Solid lines indicate first-order trend lines.
